# Viability of Airborne Tumor Cells during Excision by Ultrasonic Device

**DOI:** 10.1155/2017/4907576

**Published:** 2017-04-09

**Authors:** Masakazu Hashimoto, Tsuyoshi Kobayashi, Hirotaka Tashiro, Shintaro Kuroda, Yoshihiro Mikuriya, Tomoyuki Abe, Yuka Tanaka, Hideki Ohdan

**Affiliations:** Department of Gastroenterological and Transplant Surgery, Hiroshima University Hospital, Hiroshima, Japan

## Abstract

*Background*. Laparoscopic surgery has become more widely used, but peritoneal dissemination and port-site metastasis have been reported to occur in these surgeries. One reason for these problems is the ultrasonically activated scalpel (UAS) used for laparoscopic surgery. This study aimed to investigate the viability of airborne cells released during cancer dissection using a UAS.* Methods*. Flank tumors measuring about 2 cm were induced in male NOD-Cg-Rag1^tm1Mom^IL2rg^tm1wjl/SzJ^ mice by subcutaneous injection of 1 × 10^6^ HepG2 cells. Dissection was performed with UAS (in high or low power modes) and PowerStar bipolar scissors. The mist of released tissue was collected in cell culture medium. The viability of the cellular material was assessed with trypan blue exclusion cell counting, counting after immunofluorescence staining, and flow cytometric analysis.* Results*. Large quantities of cellular debris were trapped in the tissue dispersed by both devices. In all experiments, there were significantly more viable cells produced by the UAS in high power mode. By using suction at the excision site, the number of viable cancer cells was reduced.* Conclusions*. This study demonstrates that viable cancer cells can be released into the nearby environment during tumor ablation with a UAS.

## 1. Introduction

In recent years, surgery using a laparoscope has been compared with the traditional laparotomy, showing that its curative outcome is not inferior. Laparoscopic surgery has become less invasive due to the improvements in laparoscopic equipment and the accumulation of clinical experience. The advantages of laparoscopic surgery leave no doubt and will continue to develop in the future.

However, peritoneal dissemination and port-site metastasis were reported in laparoscopic surgery for the first time in 1978 [1] and have since been reported many times in such procedures as gastroenterological surgery [2, 3], gynecological surgery [4], and surgery of the urinary organs [5], with an incidence of almost 1%.

This problem with the laparoscopic tool is thought of as one cause of relapse, and it seems that it is necessary to further evaluate the ultrasonically activated scalpel (UAS) used to resect the tumor and the lymph nodes. Previously, there was a report that no viable carcinoma cells exist in the mist that the tool creates [6]. However, this evaluation may not be sufficient, because the viability of airborne cells was assessed only by electron micrograph.

We examined whether living cancer cells exist in the mist that is dispersed by the UAS, for the evaluation of the safety of laparoscopy-assisted surgery.

## 2. Materials and Methods

### 2.1. Cell Line and Culture Conditions

Human hepatoma cell line HepG2 was obtained from the American Type Culture Collection (ATCC). The cells were grown in Dulbecco's Modified Eagle Medium (DMEM; Invitrogen, Carlsbad, CA, USA) containing 10% fetal calf serum (Invitrogen, Carlsbad, CA, USA) in a humidified atmosphere of 95% air and 5% CO_2_ at 37°C.

### 2.2. Animal and Tumor Preparation

We used 20-week-old NOD-Cg-Rag1^tm1Mom^IL2rg^tm1wjl/SzJ^ mice for the tumor model. HepG2 cells were injected subcutaneously into the flank of the mice at 1 × 10^6^ cells/mouse. After about one month, the tumor size was about 2 cm and was used for the following experiment. All animal experiments were performed in accordance with the guidelines set by the United States National Institutes of Health (1996).

### 2.3. Collection of Dispersed Cells

The tumor was excised directly by UAS using Harmonic Laparoscopic Coagulation Shears (LCS) (Johnson and Johnson, New Brunswick, NJ, USA) and PowerStar scissors (Johnson and Johnson, New Brunswick, NJ, USA). This instrument was used in high power mode (“LCS high”), high power mode with a suction unit (“LCS high + suction”), and low power mode (“LCS low”), and these were compared to excision using PowerStar bipolar scissors (“PowerStar”). Resection of the tumor mass, which was excised from mice, was performed in a 50 mL Falcon tube and the mist was collected in 3 mL of DMEM, and they were isolated to three experiments system. As a control, the same procedure was conducted using the cultured cell line. After collection, samples were stored at 37°C to ensure that the cell survival rate would not decrease and used for the following procedure as quickly as possible.

### 2.4. Cell Counting

#### 2.4.1. Viable Cell Counting

The number of viable cells was determined by trypan blue dye exclusion using a microscope.

#### 2.4.2. Immunofluorescence Staining

A cover glass previously coated with fibronectin was placed in a 35 mm dish with DMEM, and the surgical mist that had been collected was added. After incubation overnight, the tumor cells were fixed in 4% paraformaldehyde and then washed with PBS and permeabilized in 0.1% Tween-20 (Sigma-Aldrich, St. Louis, MO, USA). After being blocked with 10% bovine serum albumin, the cells were incubated with anti-Ep-CAM antibody (EBA1) (Santa Cruz Biotechnology Inc., Dallas, TX, USA), which was detected with fluorochrome-coupled secondary antibody (Alexa Fluor 488 Goat Anti-Mouse IgG; Molecular Probes Invitrogen, Carlsbad, CA, USA). The cells were observed under a fluorescence microscope (BZ-8000) and analyzed with BZ-II Analyzer software (KEYENCE Japan, Osaka, Japan).

### 2.5. Flow Cytometric Analysis

The number of cancer cells generated in a mist among the 2.0 × 10^4^ viable cells collected previously was determined by flow cytometry. To distinguish the epithelial cells from blood cells, HepG2 cells were stained with anti-Ep-CAM antibody and anti-CD45 FITC (eBioscience, Inc., San Diego, CA, USA) monoclonal antibodies. We counted the number of cells stained for Ep-CAM. Dead cells were excluded from the analysis by propidium iodide staining. All analyses were performed using a FACSCalibur cytometer (BD Bioscience, Mountain View, CA, USA).

### 2.6. Statistical Analysis

Quantitative data are represented as the mean ± SE. Comparisons between data within the same experiments were analyzed using the Student's *t*-test. A *p* value of <0.05 was considered statistically significant. All statistical analyses were performed using the JMP 10 software for Windows (SAS Institute Japan).

## 3. Results

### 3.1. Quantification of Viable Cells from Tumor Excision

**Figure 1:**
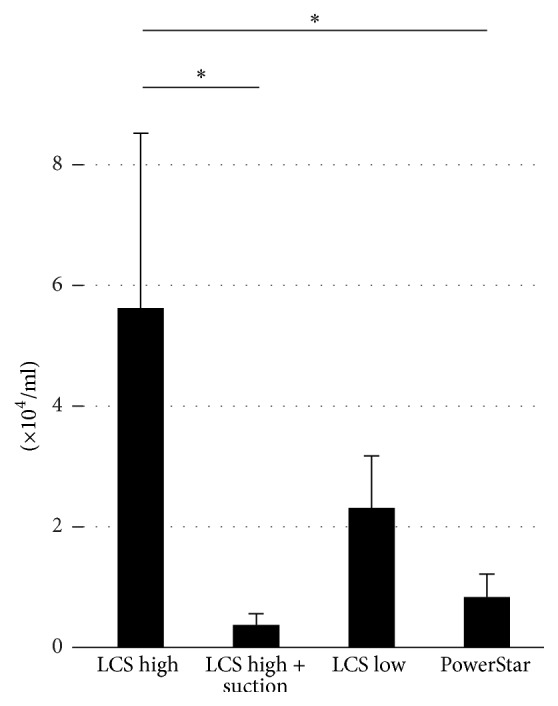
Trypan blue cell counting. In LCS high, LCS high + suction, LCS low, and PowerStar conditions, viable cancer cells were detected. The LCS high method resulted in significantly more viable cancer cells in comparison with LCS high + suction or PowerStar (^*∗*^*p* < 0.05).

Each tumor was excised with LCS high, LCS high + suction, LCS low, and PowerStar methods, and the tumor cells from the site were collected and counted (Figure 1). LCS high excision resulted in 5.6 ± 2.9 × 10^4^ cells/mL, LCS high + suction in 0.4 ± 0.2 × 10^4^ cells/mL, LCS low in 2.3 ± 0.9 × 10^4^ cells/mL, and PowerStar in 0.8 ± 0.4 × 10^4^ cells/mL. When removing the tumor using LCS high, a large amount of cell-containing mist was generated and thus many tumor cells were collected.

### 3.2. Immunofluorescence Staining

**Figure 2:**
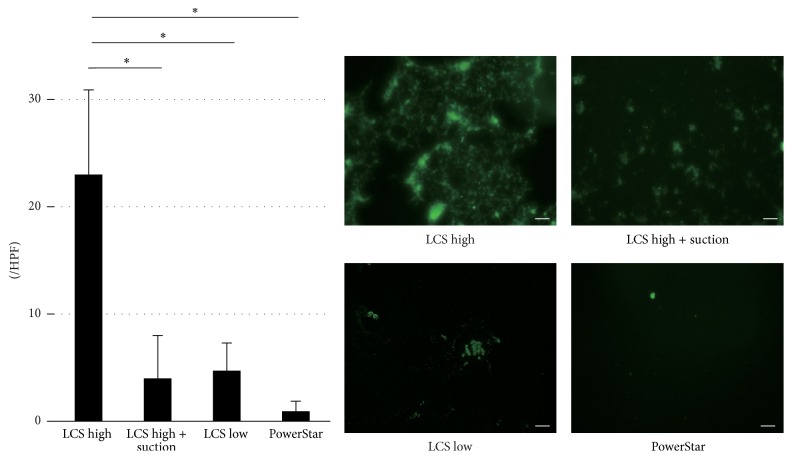
Immunofluorescence staining (anti-Ep-CAM antibody). In LCS high, LCS high + suction, LCS low, and PowerStar methods, viable cancer cells were detected. The LCS high resulted in significantly more viable cancer cells in comparison with LCS high + suction, LCS low, or PowerStar (^*∗*^*p* < 0.05). The white bar is 50 *µ*m.

The number of cells excised by LCS high was significantly greater than that by LCS high + suction, LCS low, and Power Star methods. Based on the number of viable cells released, the methods can be ordered: LCS high (28.9 ± 8.7 cells/high power field [HPF]), LCS high + suction (8.0 ± 8.0 cells/HPF), LCS low (2.7 ± 1.9 cells/HPF), and PowerStar (0.7 ± 0.3 cells/HPF) (Figure 2).

### 3.3. Flow Cytometric Analysis

**Figure 3:**
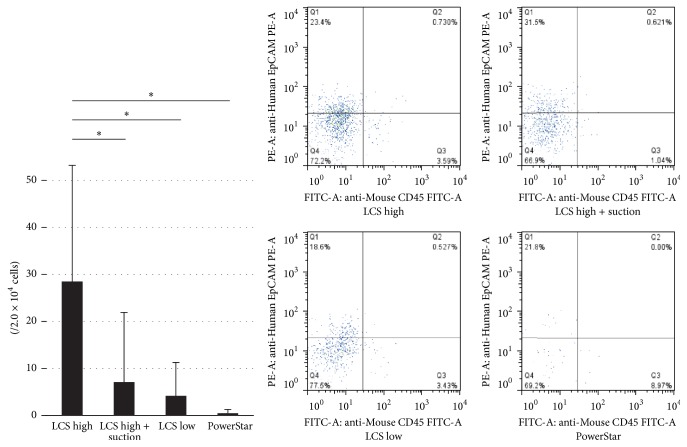
Flow cytometric analysis (anti-Ep-CAM antibody). In LCS high, LCS high + suction, LCS low, and PowerStar conditions, viable cancer cells were detected. In each group, there were significant differences (^*∗*^*p* < 0.05).

There was a significant difference between the number of cells obtained by LCS high and LCS high + suction, and between LCS high + suction and powerstar: LCS high: (28.5 ± 24.7/2.0 × 10^4^ cells), LCS high + suction: (7.1 ± 14.8/2.0 × 10^4^ cells), LCS low: (4.2 ± 7.1/2.0 × 10^4^ cells), and powerstar: (0.5 ± 0.8/2.0 × 10^4^ cells) (Figure 3).

## 4. Discussion

This study confirmed that carcinoma cells existed in the mist generated when a UAS (LCS) was used to remove the tumor. We also showed that cell viability and cell adhesion were preserved in these cells.

Previously, it was shown that these cells were unable to survive because of damage to the cell membrane. This was shown by electron microscopy, in a study on the existence of carcinoma cells in surgical mist examined in vitro [6]. We proved that living cells exist among the dispersed material by three methods in the current study. Immunofluorescence staining sufficiently confirmed cell viability and adhesive ability. Moreover, when observed over time, these cells had a proliferation rate similar to the original cell line (data not shown). The number of carcinoma cells was determined by the exclusion of dead cells and blood cells, using a flow cytometric method. In every detection and counting method we used, there was a similar result, which is the fact that the LCS high power excision method results in a danger of scattering many carcinoma cells.

In a clinical study, a randomized controlled study with a mean follow-up of 21 months compared port-site metastases and recurrence rates of laparoscopically resected colon carcinomas (44 cases) with conventional open colectomies (47 cases). This study showed no wound- or port-site metastases, with similar recurrence rates in both groups: 16.1% versus 15% [7]. The rate of peritoneal dissemination associated with port-site metastasis is not increased by the use of laparoscopic procedures, as reported in a randomized trial comparing laparoscopic-assisted colectomy and open colectomy for the treatment of nonmetastatic colon cancer [8]. This means that the dissemination is related to the advanced nature and the biological behavior of the tumor, rather than to the laparoscopic technique itself [9, 10].

However, there are some hypotheses for the mechanism by which peritoneal dissemination and port-site metastasis happens, and Castillo and Vitagliano reported that the following may all play a role: (a) tumor-related factors, (b) wound-related factors (including the immune response), and (c) surgery-related factors [11]. Depressed immune function may also contribute to tumor recurrence and metastasis. Overall immune function is diminished in the perioperative period because of several mediating factors, including anesthetic agents, opioids, surgical trauma, blood transfusions, temperature changes, pain, and psychological stress [12]. In animal models, surgical trauma has been shown to reduce natural killer cell activity and promote tumor metastasis [12, 13]. It was thought that these clinical reports may not apply to laparoscopic surgery, which results in a smaller wound and could maintain immunity.

UAS were first introduced and widely used in laparoscopic surgery and robot assisted surgery, and they are a quite useful surgical apparatus in the surgical field [14, 15]. UAS is effective in not only laparoscopic surgery but also conventional open surgery. Advantages of UAS over conventional electrosurgical instruments include reduced operating times, less intraoperative blood loss, and reduced leakage from the cut surface of organs in a variety of surgeries such as cholecystectomy, thyroidectomy, gastrectomy for gastric cancer, colorectal surgery, and pancreatic and hepatic resections [16–22]. It seems that it is important to understand the characteristics of this device used regardless of the type of surgery; this is true in laparoscopic surgery or laparotomy.

As a method for preventing dissemination, we showed that we can decrease the number of cells by changing from the high power mode of the LCS to low power and by using suction adjacent to the excision site. In clinical settings, when excising a tumor, it is thought that the number of carcinoma cells that are dispersed into the intraperitoneal cavity can be decreased by reducing the power used or using suction.

The present study had several limitations. Avoiding direct manipulation of the tumor, which is the main precaution surgeons must take during oncologic surgery, was not considered in this study. This study focused only on the airborne viable cancer cells released during cutting of tumors using LCS, not on clinical practice in detail. We believe that further studies, using animal models, are needed.

## 5. Conclusions

In conclusion, this study demonstrates that airborne viable cancer cells can be released during tumor ablation with the UAS. Moreover, it was shown that the number of cells released can be decreased by changing from the high power mode of the LCS to the low power mode and by applying suction adjacent to the excision site.

## References

[1] Dobronte Z., Wittman T., Karacsony G. (1978). Rapid development of malignant metastases in the abdominal wall after laparoscopy. *Endoscopy*.

[2] Russi E. G., Pergolizzi S., Mesiti M. (1992). Unusual relapse of hepatocellular carcinoma. *Cancer*.

[3] Le Moine M.-C., Navarro F., Burgel J. S. (1998). Experimental assessment of the risk of tumor recurrence-after laparoscopic surgery. *Surgery*.

[4] Ramirez P. T., Frumovitz M., Wolf J. K., Levenback C. (2004). Laparoscopic port-site metastases in patients with gynecological malignancies. *International Journal of Gynecological Cancer*.

[5] Rassweiler J., Tsivian A., Ravi Kumar A. V. (2003). Oncological safety of laparoscopic surgery for urological malignancy: experience with more than 1,000 operations. *Journal of Urology*.

[6] Nduka C. C., Poland N., Kennedy M., Dye J., Darzi A. (1998). Does the ultrasonically activated scalpel release viable airborne cancer cells?. *Surgical Endoscopy*.

[7] Lacy A. M., Delgado S., García-Valdecasas J. C. (1998). Port site metastases and recurrence after laparoscopic colectomy: a randomized trial. *Surgical Endoscopy*.

[8] Lacy A. M., García-Valdecasas J. C., Delgado S. (2002). Laparoscopy-assisted colectomy versus open colectomy for treatment of non-metastatic colon cancer: a randomised trial. *Lancet*.

[9] Ramos J. M., Gupta S., Anthone G. J., Ortega A. E., Simons A. J., Beart R. W. (1994). Laparoscopy and colon cancer: is the port site at risk? A preliminary report. *Archives of Surgery*.

[10] Cook T. A., Dehn T. C. B. (1996). Port-site metastases in patients undergoing laparoscopy for gastrointestinal malignancy. *British Journal of Surgery*.

[11] Castillo O. A., Vitagliano G. (2008). Port site metastasis and tumor seeding in oncologic laparoscopic urology. *Urology*.

[12] Vallejo R., Hord E. D., Barna S. A., Santiago-Palma J., Ahmed S. (2003). Perioperative immunosuppression in cancer patients. *Journal of Environmental Pathology, Toxicology and Oncology*.

[13] Eggermont A. M. M., Steller E. P., Sugarbaker P. H. (1987). Laparotomy enhances intraperitoneal tumor growth and abrogates the antitumor effects of interleukin-2 and lymphokine-activated killer cells. *Surgery*.

[14] Amaral J. F. (1994). The experimental development of an ultrasonically activated scalpel for laparoscopic use. *Surgical Laparoscopy & Endoscopy*.

[15] Amaral J. F. (1995). Laparoscopic cholecystectomy in 200 consecutive patients using an ultrasonically activated scalpel. *Surgical Laparoscopy & Endoscopy*.

[16] Bessa S. S., Abdel-Razek A. H., Sharaan M. A., Bassiouni A. E., El-Khishen M. A., El-Kayal E.-S. A. (2011). Laparoscopic cholecystectomy in cirrhotics: a prospective randomized study comparing the conventional diathermy and the harmonic scalpel for gallbladder dissection. *Journal of Laparoendoscopic and Advanced Surgical Techniques*.

[17] Hubner M., Demartines N., Muller S., Dindo D., Clavien P.-A., Hahnloser D. (2008). Prospective randomized study of monopolar scissors, bipolar vessel sealer and ultrasonic shears in laparoscopic colorectal surgery. *British Journal of Surgery*.

[18] Voutilainen P. E., Haglund C. H. (2000). Ultrasonically activated shears in thyroidectomies: a randomized trial. *Annals of Surgery*.

[19] Tanaka T. (2002). Use of ultrasonically activated shears improves the safety of pancreaticojejunostomy after pancreaticoduodenectomy. *Archives of Surgery*.

[20] Belli G., Limongelli P., Belli A. (2008). Ultrasonically activated device for parenchymal division during open hepatectomy. *HPB*.

[21] Choi M.-G., Oh S. J., Noh J. H., Sohn T. S., Kim S., Bae J. M. (2014). Ultrasonically activated shears versus electrocautery in open gastrectomy for gastric cancer: a randomized controlled trial. *Gastric Cancer*.

[22] Sugo H., Mikami Y., Matsumoto F., Tsumura H., Watanabe Y., Futagawa S. (2001). Comparison of ultrasonically activated scalpel versus conventional division for the pancreas in distal pancreatectomy. *Journal of Hepato-Biliary-Pancreatic Surgery*.

